# Incorporating ChatGPT in Medical Informatics Education: Mixed Methods Study on Student Perceptions and Experiential Integration Proposals

**DOI:** 10.2196/51151

**Published:** 2024-03-20

**Authors:** Sabrina Magalhães Araujo, Ricardo Cruz-Correia

**Affiliations:** 1 Center for Health Technology and Services Research Faculty of Medicine University of Porto Porto Portugal; 2 Department of Community Medicine, Information and Decision Sciences Faculty of Medicine University of Porto Porto Portugal; 3 Working Group Education European Federation for Medical Informatics Le Mont-sur-Lausanne Switzerland

**Keywords:** education, medical informatics, artificial intelligence, AI, generative language model, ChatGPT

## Abstract

**Background:**

The integration of artificial intelligence (AI) technologies, such as ChatGPT, in the educational landscape has the potential to enhance the learning experience of medical informatics students and prepare them for using AI in professional settings. The incorporation of AI in classes aims to develop critical thinking by encouraging students to interact with ChatGPT and critically analyze the responses generated by the chatbot. This approach also helps students develop important skills in the field of biomedical and health informatics to enhance their interaction with AI tools.

**Objective:**

The aim of the study is to explore the perceptions of students regarding the use of ChatGPT as a learning tool in their educational context and provide professors with examples of prompts for incorporating ChatGPT into their teaching and learning activities, thereby enhancing the educational experience for students in medical informatics courses.

**Methods:**

This study used a mixed methods approach to gain insights from students regarding the use of ChatGPT in education. To accomplish this, a structured questionnaire was applied to evaluate students’ familiarity with ChatGPT, gauge their perceptions of its use, and understand their attitudes toward its use in academic and learning tasks. Learning outcomes of 2 courses were analyzed to propose ChatGPT’s incorporation in master’s programs in medicine and medical informatics.

**Results:**

The majority of students expressed satisfaction with the use of ChatGPT in education, finding it beneficial for various purposes, including generating academic content, brainstorming ideas, and rewriting text. While some participants raised concerns about potential biases and the need for informed use, the overall perception was positive. Additionally, the study proposed integrating ChatGPT into 2 specific courses in the master’s programs in medicine and medical informatics. The incorporation of ChatGPT was envisioned to enhance student learning experiences and assist in project planning, programming code generation, examination preparation, workflow exploration, and technical interview preparation, thus advancing medical informatics education. In medical teaching, it will be used as an assistant for simplifying the explanation of concepts and solving complex problems, as well as for generating clinical narratives and patient simulators.

**Conclusions:**

The study’s valuable insights into medical faculty students’ perspectives and integration proposals for ChatGPT serve as an informative guide for professors aiming to enhance medical informatics education. The research delves into the potential of ChatGPT, emphasizes the necessity of collaboration in academic environments, identifies subject areas with discernible benefits, and underscores its transformative role in fostering innovative and engaging learning experiences. The envisaged proposals hold promise in empowering future health care professionals to work in the rapidly evolving era of digital health care.

## Introduction

Generative pre-trained transformers have evolved into potent language models with diverse education applications, including personalized and problem-based learning that emphasizes critical thinking [1]. They offer a chat interface for natural interactions, which can be valuable in engaging students in educational discussions. Additionally, these models can be adjusted to align with specific educational objectives and generate text embeddings, enabling tasks such as classification, recommendations, and similarity analysis. Furthermore, their accessibility through application programming interfaces (APIs) opens the door to integrate them into various educational applications beyond chat interfaces.

The integration of these artificial intelligence (AI) technologies, including ChatGPT, into the educational environment has the potential to improve the student learning experience [1-6]. By incorporating ChatGPT into the teaching and learning process in higher education, students can be supported throughout their educational journey and develop the necessary skills to effectively use AI in professional settings [4,7,8].

Integrating ChatGPT into the field of medical informatics education could not only enrich the learning experience but also empower students to apply AI skills, preparing them to tackle the complex challenges they will encounter in their future careers in health care [9]. For instance, professionals in medical informatics can use AI in developing decision support systems for diagnosing medical images and predicting patient outcomes based on data analysis. These applications demonstrate how a strong foundation in AI, facilitated by ChatGPT, can enhance the capabilities of future medical informatics professionals in delivering quality health care services.

Medical informatics, commonly referred to as biomedical or health informatics, is an umbrella term [10,11] that encompasses the use of information and communication technologies in health care. It is a fundamental field of study that caters to a diverse range of disciplines [12]. The field concentrates on using biomedical data, information, and knowledge for scientific inquiry, problem-solving, and decision-making endeavors that aim to advance care quality and delivery [13]. There is a growing interest in biomedical and health informatics (BMHI) education [14] due to the increasing demand for professionals who can address BMHI issues through the development, implementation, and evaluation of innovative technological solutions [15].

The educational requirements for BMHI vary depending on the level of education and career progression, with different pedagogical approaches needed to provide theoretical knowledge, practical skills, and a mature attitude [16]. Although medical informatics knowledge is globally applicable and requires international standards, education in this field is typically localized, with competencies being tailored to the specific environment in which they will be used [17]. Variations in educational and health care systems result in differences in BMHI education across countries. Nevertheless, despite this variability, fundamental similarities can be identified and used as a framework for recommendations [16,18].

The International Medical Informatics Association (IMIA), through its educational recommendations, has outlined essential knowledge domains for teaching in the field of BMHI, including the domain of computer science, data, and information [19]. Within this domain, there is a particular emphasis on imparting students with a deep understanding of the fundamental principles underlying emerging technologies, such as AI [19]. Notably, the most recent IMIA recommendation signifies the first explicit inclusion of AI as a topic within a BMHI knowledge domain. These IMIA recommendations offer a valuable framework for the development of educational programs, enabling the integration of essential competencies in medical informatics into the curricula of undergraduate medicine programs and master programs in the field, for instance. However, there is currently a dearth of specific recommendations regarding the inclusion of AI skills in the curriculum [9].

Since its launch, ChatGPT has ignited discussions surrounding its application in education. Rather than outright banning its use in universities, this presents an opportune moment to reassess teaching methodologies and examination practices in higher education, with the goal of preparing students for the digital world [2,3,5,7,20,21]. ChatGPT represents the initial step of a broader trend, and we must adapt to collaborate with it instead of opposing its presence [22]. In the education domain, ChatGPT will be able to offer interactive and personalized learning experiences, accessible on various devices. It can speed up routine tasks like assessments, allowing professors more time for personalized teaching. Furthermore, it can generate diverse educational content, ensure round-the-clock availability, assist in language learning, and promote innovative teaching methods.

It is crucial for faculty, professors, and students to be cognizant of both the potential and limitations of ChatGPT while also addressing ethical concerns [1,4,7,23,24] and ensuring accessibility in its implementation within academic settings. Encouraging the integration of ChatGPT in education requires the formulation of policies that promote best practices, nurturing students’ critical thinking and equipping them with the necessary skills to effectively use AI tools [4,7,25]. To foster the development of critical thinking, engaging students in activities that prompt them to verify the accuracy, veracity, and potential biases of the text generated by ChatGPT is essential [2,26,27].

Building upon the aforementioned discussions and considerations, this study aims to contribute meaningfully to the broader objective of integrating AI education within the field of medical informatics. Recognizing the significant relevance of ChatGPT as an AI tool to the medical informatics courses offered in the master’s programs in medicine and medical informatics at the Faculty of Medicine of the University of Porto (FMUP) in Portugal, this research seeks to address a proposal for integration of ChatGPT in the educational process. The first objective is to compile the opinions of students enrolled in all levels of the medical faculty’s programs with the aim of obtaining a general perception of their perspectives and experiences regarding the use of ChatGPT as an educational tool. Furthermore, this study endeavors to provide practical proposals for professors, offering examples of prompts for incorporating ChatGPT into their teaching activities, in order to enhance the educational benefits for students in medical informatics courses.

## Methods

### Ethical Considerations

A structured questionnaire was designed and submitted to the ethics committee of Faculty of Medicine of the University of Porto (105/CEFMUP/2023) to ensure ethical considerations in conducting this research and obtain approval for data collection. Students who participated in the questionnaire were explicitly informed that their participation in the research was entirely voluntary and were assured of confidentiality and anonymity regarding their responses.

### Participants and Questionnaire

This study used a mixed methods approach involving students enrolled at all levels of the medical faculty’s programs. The survey aimed to provide initial insight into medical informatics students’ perspectives regarding the use of ChatGPT in teaching. The exploratory survey served as a preliminary assessment to outline proposals for incorporating the tool classes.

The questionnaire consisted of a total of 25 questions, comprising both closed-ended and open-ended formats, which were electronically distributed to 105 students enrolled in programs at the FMUP that offer medical informatics courses. The closed-ended questions aimed to assess the participants’ familiarity with ChatGPT, their perception of the technology’s use in educational contexts, and their attitudes toward using the application in academic and learning tasks. Participants were asked to indicate their level of agreement on a Likert scale, ranging from “strongly agree” to “strongly disagree,” enabling nuanced responses.

In parallel, the open-ended questions encouraged participants to provide comprehensive and detailed feedback, sharing specific instances of their interactions and experiences with ChatGPT.

### Data Analysis

The data collected from the questionnaire were analyzed using descriptive statistical techniques to summarize the quantitative responses. Thematic analysis was used to identify recurring themes and patterns in the qualitative responses, providing deeper insights into the students’ perceptions and suggestions.

### Literature Review and Description of Course Learning Outcomes

The methodology of this study also involved conducting a comprehensive literature review to explore the current publications pertaining to the implementation of AI in higher education settings. Specifically, the focus was on examining the integration of ChatGPT within the context of teaching medical informatics and assessing its alignment with international recommendations for effective pedagogy in this domain.

To assess the potential benefits of incorporating ChatGPT into educational practices, the study describes the learning outcomes of 2 proposed courses, carefully designed based on the competencies and skills expected of medical informatics students. The authors of this research, along with other esteemed members of the faculty, collaboratively deliberated on the proposals for using ChatGPT, aiming to optimize its functionalities both in master’s programs in medicine and medical informatics.

In addition, it is expected to exemplify specific prompts to be used by students and professors to maximize the tool’s potential to facilitate learning experiences. These prompts are carefully designed to engage students in critical thinking, problem-solving, and knowledge exploration while also aiding professors in delivering exemplary instruction.

## Results

### Questionnaire

In July 2023, the questionnaire was distributed to the students through their institutional email addresses. Out of the recipients, a noteworthy 25 university students actively participated by responding to the survey, resulting in a response rate of approximately 24%. The majority of respondents identified as male, accounting for 56% (n=14) of the total sample, with an average age of 35.2 (SD 8.6) years. Table 1 provides a concise summary of the key demographic characteristics of the participating students.

**Table 1:** Characteristics of the participants (N=25).

Characteristics		Values
**Sex, n (%)**		
	Female	11 (44)
	Male	14 (56)
**Age (years)**		
	20-25, n (%)	2 (8)
	26-30, n (%)	6 (24)
	31-35, n (%)	8 (32)
	>35, n (%)	9 (36)
	Mean (SD)	35.2 (8.6)

Regarding the use of ChatGPT (Table 2), 52% (n=13) of the students indicated that they had their initial encounter with the chatbot during the second semester of 2022. Among them, a considerable proportion (n=4, 16%) reported using it on a daily basis, while the majority (several times during the week) found it to be a frequent resource. Impressively, 92% (n=23) of the students conveyed their satisfaction with the responses generated by ChatGPT, with 48% (n=12) expressing a high level of reliance on its answers and offering strong endorsements of its implementation among their peers. Nevertheless, a subset of participants (n=5, 20%) disclosed that they rarely place trust in the responses provided by the system. Furthermore, 96% (n=24) of the participants asserted that the tool comprehends the contextual intricacies of questions well. However, they noted that occasionally, to obtain the desired response, it becomes necessary to reformulate the query.

**Table 2:** Answers to questionnaire questions about the use of ChatGPT by medical faculty students (N=25).

Question and answers			Respondents, n (%)
**Do you usually talk to your colleagues about ChatGPT?**			
	Ever	11 (44)	
	Occasionally	13 (52)	
	Often	1 (4)	
**When did you first use ChatGPT?**			
	Between March and April 2023	4 (16)	
	Between January and February 2023	7 (28)	
	Between July and December 2022	13 (52)	
	Between January and June 2022	0 (0)	
	In 2021	1 (4)	
**Do you use ChatGPT regularly?**			
	Yes every day	4 (16)	
	Yes, several times a week	13 (52)	
	I use it from time to time	8 (32)	
**How satisfied are you with ChatGPT’s responses?**			
	Very satisfied	5 (20)	
	Satisfied	18 (72)	
	I have a neutral position on this	2 (8)	
**Do you trust the information provided by ChatGPT?**			
	Most of the time	12 (48)	
	Sometimes	8 (32)	
	Rarely	5 (20)	
**Does ChatGPT understand the context of your questions well?**			
	Very good	7 (28)	
	Good	17 (68)	
	No opinion	1 (4)	
**When using ChatGPT, do you have to rephrase questions to get the answers you want?**			
	Rarely	7 (28)	
	Sometimes	17 (68)	
	Often	1 (4)	
**Would you recommend ChatGPT to your colleagues?**			
	Definitely	18 (72)	
	Probably	5 (20)	
	I am not sure	1 (4)	
	Probably not	1 (4)	

Concerning attitudes toward the use of ChatGPT for learning and academic purposes (Table 3), a majority of students demonstrated openness to adopting this form of chatbot and express intentions to incorporate it regularly into their educational endeavors. Nevertheless, it is noteworthy that 8% (n=2) of the participants held a dissenting perspective and are resolutely against its use in academic activities.

**Table 3:** Attitudes toward using ChatGPT for learning and academic tasks.

Statement and Likert scale		Respondents, n (%)
**I think using a tool like ChatGPT would be a good idea to support learning**		
	Strongly agree	13 (52)
	Agree	8 (32)
	Neither agree nor disagree	4 (16)
**I will start using ChatGPT to support learning and completing academic tasks**		
	Strongly agree	8 (32)
	Agree	9 (36)
	Neither agree nor disagree	7 (28)
	Disagree	1 (4)
**I will ask my colleagues about ChatGPT and how they use it**		
	Strongly agree	7 (28)
	Agree	8 (32)
	Neither agree nor disagree	10 (40)
**I intend to create the habit of using ChatGPT to support learning and carry out my academic work**		
	Strongly agree	6 (24)
	Agree	12 (48)
	Neither agree nor disagree	5 (20)
	Disagree	2 (8)
**I will use ChatGPT or other similar chatbots whenever the opportunity arises**		
	Strongly agree	9 (36)
	Agree	11 (44)
	Neither agree nor disagree	3 (12)
	Disagree	2 (8)
**I have a bad feeling about ChatGPT and artificial intelligence in general**		
	Strongly agree	2 (8)
	Agree	1 (4)
	Neither agree nor disagree	8 (32)
	Disagree	10 (40)
	Strongly disagree	4 (16)
**In my opinion, the use of ChatGPT or similar chatbots for academic tasks should not be allowed**		
	Strongly agree	1 (4)
	Agree	1 (4)
	Neither agree nor disagree	5 (20)
	Disagree	6 (24)
	Strongly disagree	12 (48)

Regarding the perceptions of ChatGPT as an academic support tool (Table 4), a significant proportion (n=18, 72%) of students concur with the notion that the ChatGPT tool has the potential to enhance and facilitate learning experiences. Furthermore, an overwhelming majority strongly agree that its implementation streamlines the execution of tasks, promoting efficiency within the academic context.

**Table 4:** Perceptions of ChatGPT as an academic support tool.

Statement and Likert scale			Respondents, n (%)
**Using ChatGPT improves learning**			
	Strongly agree	12 (48)	
	Agree	6 (24)	
	Neither agree nor disagree	6 (24)	
	Disagree	1 (4)	
**Using the ChatGPT can make learning tasks easier to complete**			
	Strongly agree	12 (48)	
	Agree	10 (40)	
	Neither agree nor disagree	3 (12)	
**I find ChatGPT a very useful tool to support learning**			
	Strongly agree	15 (60)	
	Agree	7 (28)	
	Neither agree nor disagree	3 (12)	
**Using ChatGPT can increase my productivity as a student**			
	Strongly agree	16 (64)	
	Agree	6 (24)	
	Neither agree nor disagree	3 (12)	
**Using ChatGPT allows me to complete tasks faster**			
	Strongly agree	19 (76)	
	Agree	4 (16)	
	Neither agree nor disagree	2 (8)	

In terms of the use of ChatGPT or other AI bots in the future, the majority of responses indicate that participants find it extremely useful, especially for medical writers who are not proficient in English, as it aids in restructuring and correcting texts. Some believe that the adoption of these tools will be inevitable and increasingly common, both in academic and professional contexts, resulting in enhanced process efficiency. However, there are also ethical concerns and apprehensions regarding the potential impact on the employability of programming professionals. Moreover, participants emphasize the importance of informed use, understand the limitations, and use these tools intelligently and as complementary to specific objectives. Some express caution, recognizing that although the tools have advantages, they do not create anything new but rather help organize thoughts and facilitate a better understanding of concepts.

The responses reveal diverse opinions on the benefits and drawbacks of using AI bots like ChatGPT in education. Some students highlight the capacity to customize responses to specific questions without worrying about boring the instructor and speeding up repetitive tasks, envisioning its potential to revolutionize the educational landscape. However, there are concerns about the long-term effects and the need for caution. The AI’s biases and limitations are seen as potentially harmful to knowledge, and there are worries about the credibility of sources used and proper attribution of credits and bibliographic referencing.

**Figure 1:**
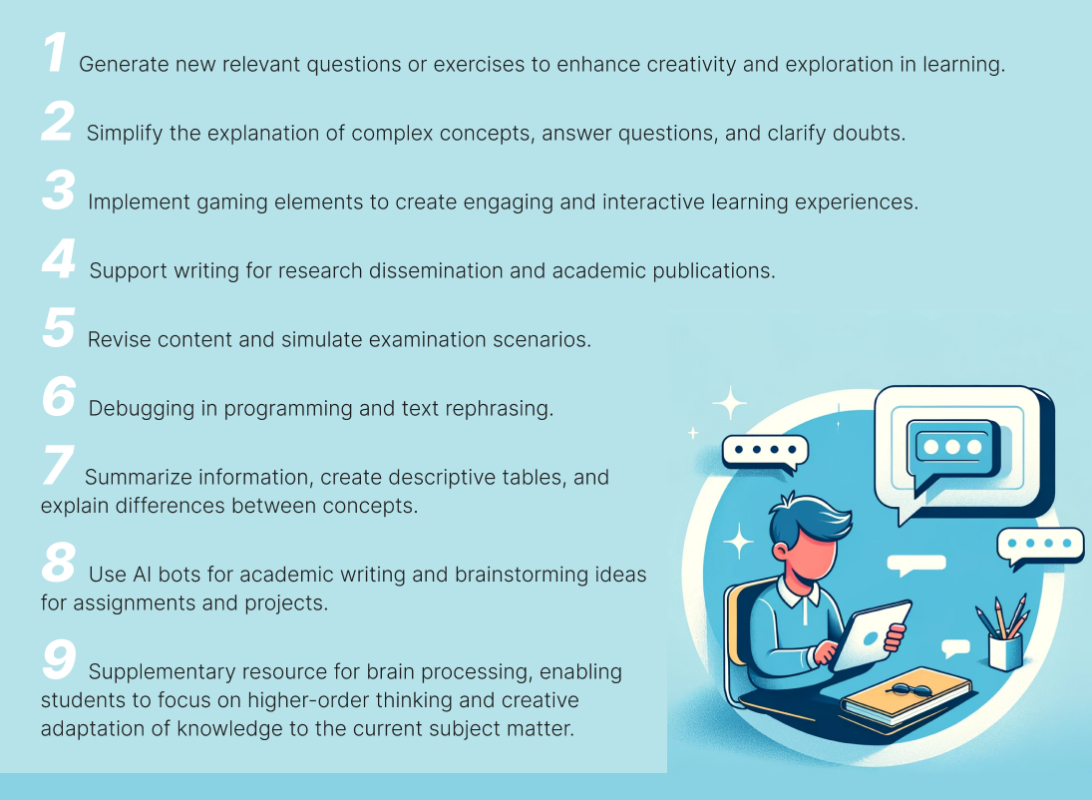
List of suggestions from medical informatics course students on how to use ChatGPT in education generated with the assistance of artificial intelligence (AI).

While some view it as a valuable tutor or assistant that is always available, others caution against its potential to promote laziness, particularly in written work, which may discourage the development of quality writing skills. Nevertheless, the quick response time for academic tasks is regarded as an advantage by some, with no apparent disadvantages seen. The major concern is the risk of a bias in thinking, whereby AI-generated ideas or responses could influence one’s own thought process, potentially inhibiting critical thinking. Despite this, many believe that ChatGPT can be a useful aid in executing certain tasks, as it does not create content but rather assists in the development and enhancement of ideas. The list of suggestions from medical informatics students on how to use ChatGPT or other AI bots in education can be seen in Figure 1.

### Integration of ChatGPT

#### Overview

The analysis of student responses to the questionnaire has revealed a positive receptiveness and interest in the use of ChatGPT as an educational tool. Building on these findings, the next phase of our investigation focused on the proposed integration of ChatGPT into classroom settings. This section explores the proposal to incorporate ChatGPT into 2 specific courses, outlining how prompts developed by professors can be applied to enhance medical informatics students’ learning experiences. Additionally, we will address the events held at the faculty to discuss and guide the implementation of ChatGPT in the context of education, emphasizing the collaboration among professors to foster educational innovation.

#### Master’s Program in Medicine

The master’s program in medicine at FMUP comprises an integrated cycle of studies totaling 360 credits, in accordance with the European Credit Transfer and Accumulation System (ECTS).

The course chosen for this study’s proposal of implementing ChatGPT in the master’s program in medicine is “DECIDES III: Decision, Data and Digital Health” (4 ECTS), which has been taught in the fourth year. By the end of this course unit, medical students are expected (1) to have knowledge and be able to discuss key topics related to health information systems and the integration of scientific evidence in health decision-making; (2) to proficiently and safely use health information systems; (3) to critically evaluate health scientific literature, particularly regarding health information systems, health technology assessment, and health decision analysis; and (4) to plan and interpret studies on health economic evaluation and decision analysis.

The course offered in the master’s program in medicine proposes the use of ChatGPT in 3 ways (Textbox 1).

**Three proposed uses of ChatGPT.**

**Assistant for simplified explanation of concepts**

ChatGPT will serve as an assistant to explain complex concepts in a simplified manner. Medical students will be encouraged to use ChatGPT outside of the classroom to enhance their understanding of various topics, including blockchain, cloud services, data quality, machine learning, electronic health records (EHRs), and mobile health. They will be prompted to request explanations in simple language. Example of prompt: *Explain the following concepts to me in a simple manner, providing examples from the healthcare field, as if I were a 16-year-old: machine learning.*

**Assistant for addressing complex problems**

ChatGPT will also be used as an assistant to address intricate problems. It will provide support to students in tackling challenging scenarios and offer insights and solutions related to medical informatics and health care. Example of prompt: *What risks and benefits has the General Data Protection Regulation brought to clinical research? For each of the risks, propose a technological solution to mitigate it. Present your findings in a table format.*

**Generation of clinical narratives and patient simulators**

This feature will enable students to simulate realistic patient cases and explore various clinical scenarios, thereby enhancing their ability to effectively and securely use health information systems with proficiency. A prompt was created, requesting that ChatGPT read the manual of a health information system used in public hospitals in Portugal and then generate a clinical case that would allow the professor to practice with the students the use of all specific functionalities of the system.

In addition to assisting students, ChatGPT will also serve as a valuable tool for professors. By using the version of ChatGPT which incorporates plugins, professors can leverage its capabilities to enhance their teaching methodologies. In Multimedia Appendix 1, an illustrative scenario of using ChatGPT is detailed, culminating in the creation of a comprehensive lesson plan for obstetrics. This includes practical exercise demonstrations, a data generator for classroom use, a compilation of clinical cases for educational purposes, and a decision tree highlighting the importance of data quality in the medical field. This setup enhances understanding of ChatGPT’s practical application in medical education, offering innovative tools for improving teaching and learning.

#### Master’s Program in Medical Informatics

The master’s program in medical informatics (MIM) comprises 120 ECTS. Established 17 years ago at FMUP, MIM recently earned accreditation from the European Federation for Medical Informatics in 2022, affirming its high quality and recognition within the field.

The MIM’s course unit proposed in this study to incorporate ChatGPT during classes in “health information systems and electronic health records” (6 ECTS), which is taught in the second semester of the master’s program.

The main objective of this course is to equip students with the necessary knowledge and skills to effectively select, design, and manage health information systems and EHRs. The course focuses on developing an understanding of health information systems, including their development and implementation processes, functions, historical evolution, the significance of shared concepts among these systems, barriers in data collection, data integration and process integration, change management, current trends in health information system development, and the main challenges and considerations related to meaningful use. By the end of the course, students are expected to have achieved specific learning outcomes and competencies in these areas.

In the MIM, the use of ChatGPT offers students a versatile tool that enhances their learning experience and skill development. By incorporating ChatGPT, students can explore new educational possibilities and engage with the technology in meaningful ways. There are 5 specific applications of ChatGPT in the course. (Textbox 2).

**Five specific applications of ChatGPT.**

**Project planning assistant**

ChatGPT will act as an assistant in project planning, providing frameworks, checklists, and real-world examples. This aims to impart project management skills crucial for the successful development and implementation of health information systems, thereby increasing the likelihood of project success and reducing system inefficiencies. Example of prompt: *We intend to develop an app to monitor asthma patients and their crises, aiding in disease self-management. What stages should the project go through from conception to commercialization?*

**Programming code generation**

Students will engage in coding exercises facilitated by ChatGPT, aimed to improve their programming skills essential in medical informatics. This personalized learning experience allows students to work at their own pace, ensuring a deeper understanding of the coding principles and practices. Example of prompt: *Generate the SQL programming code to create the database for the application.*

**Examination preparation**

ChatGPT assists students in preparing for examinations by simulating examination scenarios and providing practice questions. By inputting relevant course materials and previous examination papers, students can engage with the system to receive responses that aid their understanding of the subject matter. This feature enables students to refine their knowledge and enhance their examination performance. Instructions for students:

- Feed the chat with links to pages and PDFs containing the course content and past examinations.
- Ask ChatGPT to generate 10 examination questions. Select the 3 most interesting and well-constructed questions.
- Ask ChatGPT to generate 10 more questions similar to the chosen 3.
- Discuss with a colleague and select 2 questions each.
- Ask both respective ChatGPT models for an answer to each of the 2 questions (resulting in 4 answers).
- Compare the answers and assign a rating from 0 to 10 to each response. Ask ChatGPT to self-evaluate the answers.

**Workflow and information exploration**

ChatGPT enhances the exploration of intricate workflows and information within medical informatics. By engaging interactively with the system, students can emulate real-world health care information systems, acquiring hands-on experience in managing and analyzing complex data sets. This practical engagement deepens their comprehension of information flow dynamics and the principles of workflow optimization in a health care context.

**Technical interview preparation**

Students will prepare for technical questions likely to appear in professional interviews by leveraging ChatGPT to simulate a wide range of possible questions and scenarios. This not only aims to enhance their employability by familiarizing them with potential interview questions but also aids them in articulating their knowledge and skills effectively. As AI and machine learning continue to penetrate various aspects of health care and medical research, the ability to interact and extract meaningful insights from these systems will be a strong asset. Hence, students will not only leave the course well-prepared for interviews but also well-equipped for a future job market that demands adeptness in working with intelligent systems like ChatGPT.

Additionally, several practical exercises are planned to further enhance the learning experience in medical informatics. These include a data privacy challenge—students can be tasked with identifying potential vulnerabilities in a mock EHR system and proposing solutions to improve data privacy. There will also be an API integration exercise—students can work on connecting a health monitoring device to an existing EHR system using APIs, thereby gaining hands-on experience in system interoperability. Finally, there will be a machine learning mini-project—students are tasked with a simplified predictive modeling challenge, where they need to forecast patient outcomes using a specified set of features. They will use a popular programming language and a statistical software library to construct and assess their models, gaining practical experience in predictive analytics within a health care setting.

These exercises are designed to not only improve technical skills but also to cultivate a mindset of problem-solving and practical application in the realm of medical informatics.

#### Fostering Collaborative Efforts

As ChatGPT is a recent tool, gaining insights into students’ perceptions regarding its use in education is essential. Moreover, it is imperative to foster institutional collaboration to empower professors in effectively integrating AI tools into the teaching process. Throughout the course of several months, a series of pioneering initiatives unfolded at FMUP, spearheading the integration of ChatGPT into medical informatics education. The journey commenced in January 2023, with the introduction of ChatGPT theme in the MIM classes, providing students with a discussion about the tool’s potential application in the health care sector. As the momentum grew, a presentation was held in March 2023, engaging faculty members and researchers in exploring the practical use of ChatGPT in education.

Building on this foundation, the month of May 2023 witnessed the event “ChatGPT: Challenges for Education and Research,” orchestrated in collaboration with FMUP’s ethics committee. Subsequently, in a grander gathering titled “ChatGPT: Learning Models for Higher Education,” diverse faculty members united from disciplines ranging from engineering and arts to psychology and sciences. Together with the participation of the ethics committee chairman and the university’s vice-rector, professors from several faculties presented proposals for the incorporation of ChatGPT in classes, paving the way for the implementation of ChatGPT in the upcoming academic year.

By July 2023, the momentum reached new heights during the FMUP Summer School, with a captivating workshop focused on harnessing ChatGPT’s potential through the design of improved prompts, ensuring more profound and effective responses. As we delve into the results of these collaborative efforts, this section sought to describe notable proposals for integrating ChatGPT into medical informatics courses, as presented and discussed at the previously mentioned events.

## Discussion

### Principal Findings

Building on the proposal to integrate AI into medical programs to prepare students for their future use of such tools in professional contexts [5,8,28,29], the implementation of ChatGPT has emerged as a potentially transformative force in medical education [30,31], offering support to students in their learning journey [30,32]. The questionnaire administered to medical faculty students provided valuable insights into their perspectives and experiences with ChatGPT, shedding light on their attitudes, preferences, and intentions regarding the incorporation of AI chatbots in educational environments. The participants, with a mean age of approximately 35 (SD 8.6) years, predominantly comprised master’s and doctoral students, indicating a higher participation rate from these groups compared to undergraduate medical students. Engaging in frequent discussions with peers about ChatGPT, most participants were introduced to the tool during its initial launch in 2022. Remarkably, a majority of students used ChatGPT regularly for diverse purposes, including report writing, idea brainstorming, and text rewriting.

In general, students expressed satisfaction with ChatGPT’s responses, finding them to be reliable and contextually comprehensible. They recognized the educational potential of ChatGPT, highlighting its ability to facilitate the creation of relevant exercises, enhance writing skills, and foster exploration of new concepts. Drawing from these valuable insights, proposals for ChatGPT’s integration into the 2 master’s programs were developed. Additionally, existing references that offer a plethora of ideas for ChatGPT’s incorporation into medical education were also considered, ranging from personalized learning opportunities [33,34] to problem-based learning and clinical problem-solving approaches [35]. Moreover, ChatGPT can be harnessed for teaching assistance, generating case scenarios, and creating educational content such as summaries, questionnaires, and flashcards [34].

The participants also acknowledged the need for caution in its application and emphasized the importance of understanding its limitations. It is essential to be mindful that AI systems may engage in “hallucination,” a phenomenon where they fabricate facts and produce confident-sounding statements and seemingly legitimate citations that are, in reality, false, and not necessarily supported by their training data [2,36,37]. To mitigate such issues, future implementations of ChatGPT should consider raising student awareness of the possibility of AI-generated content and encouraging critical analysis of generated responses. Although students expressed openness to adopting ChatGPT, their critical analysis of potential impacts on education should be taken into consideration by professors when implementing ChatGPT in the classroom.

Privacy concerns surrounding student interactions with ChatGPT have been acknowledged in prior literature [4,7,24]. It is imperative that AI be used as an educational aid without the extraction of sensitive data, adhering to relevant data privacy regulations. Information acquired during a learner’s interactions with the AI system to acquire knowledge must be shielded from any inappropriate use [38]. Despite the recent availability of this tool, specific guidelines regarding anonymity techniques for ChatGPT’s full integration into our master’s programs and curricula have yet to be established within our academic context. Nevertheless, professors can proactively protect privacy by refraining from collecting personally identifiable information, opting for generic pseudonyms over real names, working with aggregated data, securing data transmission through encryption, implementing data retention policies with defined timeframes, restricting access to authorized personnel, and educating students on best practices for safeguarding their privacy. These measures collectively ensure adherence to privacy regulations and the preservation of the confidentiality of student interactions with ChatGPT.

Building upon the students’ recognition of both the potential and limitations of ChatGPT, it becomes evident that fostering a balanced approach to AI integration in education is paramount. It requires a concerted effort to leverage AI’s strengths while addressing its vulnerabilities. This is where the strategic organization of faculty events plays a pivotal role in shaping the future landscape of AI-driven education.

In terms of fostering collaboration in the academic environment, the strategic organization of faculty events scheduled between March and June 2023 presented a unique opportunity to facilitate the start of ChatGPT integration in the upcoming academic year. Facilitating open discussions on the integration of AI in education, including the use of tools like ChatGPT, is a pivotal undertaking within the academic realm [3]. It represents a critical step toward embracing best practices, exploring ethical considerations, and harnessing the potential of AI to enhance the educational experience [4]. Such efforts require the active collaboration and engagement of all stakeholders involved in educational settings, including professors, researchers, and experts in the field [4]. By fostering a collective dialogue, universities can pave the way for the effective and responsible incorporation of AI technologies into teaching and learning environments, ultimately benefiting students and shaping the future of education.

In the field of medical informatics, the development of skill-based curricula becomes indispensable to meet the complexities of health care delivery and market demands [39]. Sapci and Sapci [9] have put forth a framework for specialized AI training in medical and health informatics education, and our study’s proposals regarding the use of ChatGPT align with some of the competencies outlined in their research. For medical students, AI competencies include the application of predictive AI techniques to enhance health care efficiency and the critical evaluation of AI tools. In the case of medical informatics students, the competencies encompass the adept application of suitable machine learning algorithms to analyze intricate medical data, the seamless integration of data analytics into innovative clinical informatics systems and applications, and the formulation of data-related queries to visualize large data sets. For students pursuing computer science, the focus lies on developing programming languages tailored to address complex medical challenges [9].

Through the integration of ChatGPT into the master’s program in medicine, both students and professors will have the opportunity to harness its diverse functionalities, which play a pivotal role in promoting, for example, an understanding of complex concepts, effective problem-solving, and creating realistic medical scenarios. Consequently, the following applications of ChatGPT have been proposed for implementation: (1) acting as an assistant for simplified explanation of concepts, (2) assisting in addressing complex problems, (3) generating clinical narratives and patient simulators, and (4) enhancing teaching techniques for professors. These proposed applications hold the potential to augment the educational experience and knowledge acquisition within the field of medical informatics by medical students.

Regarding the MIM, the integration of ChatGPT is intended to offer students learning experiences that promote active engagement. Students are expected to cultivate essential skills, enhance problem-solving abilities, and equip themselves for upcoming challenges in this domain. Therefore, we have identified five specific ChatGPT applications proposed for the course: (1) project planning assistant, (2) programming code generation, (3) examination preparation, (4) workflow and information exploration, and (5) technical interview preparation. These proposed applications carry the potential to enrich the educational journey by empowering students to excel in the dynamic and evolving field of medical informatics.

### Limitations

The low number of questionnaire’s responses is a limitation of the study. However, it is important to highlight that the survey aimed to provide an initial insight into the perspectives of medical informatics students at the FMUP regarding the use of ChatGPT in teaching. The primary purpose of the survey was exploratory in nature, serving as a preliminary assessment to inform future initiatives rather than a comprehensive study with a large sample. The other limitation lies in the absence of practical implementation of the proposed ChatGPT incorporation in the current academic year. As a result, the actual impact on the teaching and learning process remains uncertain, and the benefits of AI use in medical informatics education require further empirical verification. However, the study provides valuable groundwork for future exploration and collaboration in exploring AI’s potential in education. While the ideas presented hold promise, empirical evaluation in the upcoming academic term will be imperative to ascertain their effectiveness and measure their impact on students’ learning experiences. Further research and assessment will be necessary to determine the concrete effects and refine the integration strategies. Until then, the study stands as a stepping stone for stimulating ongoing dialogue and inspiring future research endeavors in the dynamic field of AI-driven education in the teaching of medical informatics.

### Conclusions

The results of the questionnaire suggest that students perceive ChatGPT as a valuable tool for enhancing learning experiences and academic tasks, although they also emphasize the importance of informed and responsible use. The study’s findings contribute valuable insights for professors in exploring the integration of AI chatbots like ChatGPT in educational settings, with a particular focus on its suitability for medical informatics courses at master’s levels.

Additionally, the study provided a description of the learning outcomes of the 2 courses proposed for the incorporation of ChatGPT in the classroom. The collaborative efforts undertaken during 2023, including workshops and meetings with faculty members, served as pivotal moments that contributed to optimizing the use of ChatGPT as a powerful educational tool within the institution. Furthermore, specific subject areas and topics were identified as prime candidates for benefits through ChatGPT integration. The alignment of ChatGPT with these areas demonstrates its potential to increase the quality of education in the field of medical informatics.

In conclusion, the findings of this study highlight ChatGPT’s promising role in enhancing medical informatics education by equipping students and faculty with a transformative AI-driven approach. The insights gained from this research effort provide valuable prompt examples for harnessing the power of AI to create innovative educational experiences in the ever-evolving landscape of medical informatics. As we move into the era of AI-driven education, these findings hold significant implications for future pedagogical approaches, fostering an enriched learning environment that empowers the next generation of health care professionals to operate in the digital age.

## Appendix

Multimedia Appendix 1 Example of using ChatGPT to create a lesson plan for medical informatics students.

## Abbreviations

AI: artificial intelligence
API: application programming interface
BMHI: biomedical and health informatics
ECTS: European Credit Transfer and Accumulation System
EHR: electronic health record
FMUP: Faculty of Medicine of the University of Porto
IMIA: International Medical Informatics Association
MIM: master’s program in medical informatics
