# Supplementary material for: Incorporating ChatGPT in Medical Informatics Education: Mixed Methods Study on Student Perceptions and Experiential Integration Proposals
Source: JMIR Med Educ. 2024 Mar 20;10:e51151. doi: 10.2196/51151 (PMC10993110; doi:10.2196/51151)
Supplement: Multimedia Appendix 1 [file mededu_v10i1e51151_app1.pdf]

Generate a realistic dataset of patients for an exercise in which students need to study the association between Apgar scores and gestational weeks. Present the dataset in a table.

Creating a realistic dataset for studying the association between Apgar scores and gestational weeks involves generating synthetic data while maintaining the characteristics of real-world data. Here's a simplified dataset for your exercise:

```plaintext
| Patient ID | Gestational Weeks | Apgar Score |
|------------|-------------------|-------------|
| 1          | 38                | 9           |
| 2          | 39                | 8           |
| 3          | 36                | 9           |
| 4          | 40                | 7           |
| 5          | 37                | 9           |
| 6          | 38                | 8           |
| 7          | 39                | 9           |
| 8          | 35                | 7           |
| 9          | 41                | 9           |
| 10         | 38                | 8           |
| 11         | 37                | 9           |
| 12         | 39                | 9           |
| 13         | 36                | 7           |
| 14         | 40                | 8           |
| 15         | 38                | 9           |
| 16         | 39                | 8           |
| 17         | 35                | 7           |
| 18         | 41                | 9           |
| 19         | 38                | 9           |
| 20         | 37                | 8           |
```

This dataset contains 20 synthetic patient records, with each record including a unique Patient ID, Gestational Weeks (weeks of pregnancy at birth), and Apgar Score (a measure of a newborn's physical condition immediately after birth). The values in the table are randomly generated, but you can adjust them or add more records to make the dataset more extensive if needed for your exercise.

Provide an estimate of cases where the Apgar score at the 5th minute is lower than at the 1st.

To estimate the cases where the Apgar score at the 5th minute is lower than at the 1st, you can examine the dataset provided and count the instances where this condition is met. Here's the analysis:

In the dataset provided:

1. Patient 2: Apgar score at 1st minute = 8, Apgar score at 5th minute = [You can add a value here]
2. Patient 4: Apgar score at 1st minute = 7, Apgar score at 5th minute = [You can add a value here]
3. Patient 6: Apgar score at 1st minute = 8, Apgar score at 5th minute = [You can add a value here]
4. Patient 8: Apgar score at 1st minute = 7, Apgar score at 5th minute = [You can add a value here]
5. Patient 14: Apgar score at 1st minute = 8, Apgar score at 5th minute = [You can add a value here]

To estimate the number of cases where the Apgar score at the 5th minute is lower than at the 1st minute, you need to determine the values for Apgar scores at the 5th minute for the above patients. Once you have those values, count how many times the score at the 5th minute is lower than at the 1st minute.

Using this estimate, create a Python program to generate a dataset with the same initial structure, where the relationship between the Apgar scores is as you just described.

To create a Python program that generates a dataset with the same structure and the specified relationship between Apgar scores (5th minute lower than 1st minute), you can use the following code. This code uses randomization to maintain the same structure but ensures the desired relationship:

python Copy code

```
import random
import pandas as pd

# Number of patient records
num_records = 20

# Initialize an empty dataset
data = {'Patient ID': [], 'Gestational Weeks': [], 'Apgar Score 1st Minute':

for patient_id in range(1, num_records + 1):
    # Generate random gestational weeks between 35 and 41
    gestational_weeks = random.randint(35, 41)

    # Generate a random Apgar score at 1st minute between 5 and 9
    apgar_1st_minute = random.randint(5, 9)

    # Ensure that the Apgar score at 5th minute is lower than at 1st minute
    apgar_5th_minute = random.randint(0, apgar_1st_minute - 1)

    # Append data to the dataset
    data['Patient ID'].append(patient_id)
    data['Gestational Weeks'].append(gestational_weeks)
    data['Apgar Score 1st Minute'].append(apgar_1st_minute)
    data['Apgar Score 5th Minute'].append(apgar_5th_minute)

# Create a DataFrame from the generated data
df = pd.DataFrame(data)

# Print the DataFrame
print(df)
```

This code will create a dataset with the same structure as before, but this time it ensures that the Apgar score at the 5th minute is lower than at the 1st minute for each patient. You can adjust the number of patient records (`num_records`) and the ranges for gestational weeks and Apgar scores as needed.

Modify the code to save the dataset in a CSV file and the variable descriptions in an SPSS syntax file.

To modify the code to save the dataset in a CSV file and create an SPSS syntax file with variable descriptions, you can use the following code:

python Copy code

```
import random
import pandas as pd

# Number of patient records
num_records = 20

# Initialize an empty dataset
data = {'Patient ID': [], 'Gestational Weeks': [], 'Apgar Score 1st Minute':

# Variable descriptions for SPSS syntax file
variable_descriptions = {
    'Patient ID': 'Patient Identifier',
    'Gestational Weeks': 'Weeks of pregnancy at birth',
    'Apgar Score 1st Minute': 'Apgar Score at 1st Minute',
    'Apgar Score 5th Minute': 'Apgar Score at 5th Minute (Lower than 1st Min
}

for patient_id in range(1, num_records + 1):
    # Generate random gestational weeks between 35 and 41
    gestational_weeks = random.randint(35, 41)

    # Generate a random Apgar score at 1st minute between 5 and 9
    apgar_1st_minute = random.randint(5, 9)

    # Ensure that the Apgar score at 5th minute is lower than at 1st minute
    apgar_5th_minute = random.randint(0, apgar_1st_minute - 1)
```

```
    # Append data to the dataset
    data['Patient ID'].append(patient_id)
    data['Gestational Weeks'].append(gestational_weeks)
    data['Apgar Score 1st Minute'].append(apgar_1st_minute)
    data['Apgar Score 5th Minute'].append(apgar_5th_minute)

# Create a DataFrame from the generated data
df = pd.DataFrame(data)

# Save the dataset to a CSV file
df.to_csv('patient_data.csv', index=False)

# Create an SPSS syntax file for variable descriptions
with open('variable_descriptions.sps', 'w') as sps_file:
    for variable, description in variable_descriptions.items():
        sps_file.write(f"VARIABLE LABELS {variable} '{description}'.\n")

print("Dataset and SPSS syntax files saved successfully.")
```

This code generates the dataset, saves it as "patient_data.csv," and creates an SPSS syntax file named "variable_descriptions.sps" with variable descriptions. You can customize the file names as needed.

Create a LaTeX report in which you describe the prompts used to create this code. Include the Python code within the content of the report.

Creating a LaTeX report requires using a LaTeX editor like TeXShop, Overleaf, or any other LaTeX-compatible editor. Below is an example of a LaTeX report that describes the prompts used to create the Python code and includes the Python code within the content of the report. You can copy this LaTeX code into your LaTeX editor and compile it to generate a PDF report.

latex   Copy code

```latex
\documentclass{article}
\usepackage{listings} % For including code snippets
\usepackage{geometry} % For adjusting page margins
\usepackage{hyperref} % For hyperlinks

% Adjust page margins if needed
\geometry{top=1in, bottom=1in, left=1in, right=1in}

\title{Creating a Synthetic Dataset and SPSS Syntax File}
\author{Your Name}
\date{\today}

\begin{document}

\maketitle

\section{Introduction}
In this report, we will describe the prompts used to create a Python program

\section{Python Code}

Below is the Python code used to create the dataset and SPSS syntax file:

\lstinputlisting[language=Python, caption={Python code to generate the datas

\section{Description of the Code}

The Python code in Listing~\ref{lst:python_code} accomplishes the following

\begin{enumerate}
    \item It initializes an empty dataset and variable descriptions.
    \item It generates synthetic patient records with random gestational wee
    \item It ensures that the Apgar score at the 5th minute is lower than at
    \item It saves the dataset as \texttt{patient\_data.csv}.
    \item It creates an SPSS syntax file, \texttt{variable\_descriptions.sps
\end{enumerate}

\section{Conclusion}

The Python code presented in this report generates a synthetic dataset and a

For further details and the complete code, please refer to the attached Pyth

\section{Attachments}
```

```
\begin{itemize}
    \item Python Code: \texttt{your\_python\_code.py}
\end{itemize}

\end{document}
```

Make sure to replace `your_python_code.py` with the actual filename of your Python code containing the dataset generation and SPSS syntax creation logic. You can customize the report's title, author name, and any other details as needed. Compile this LaTeX document using your preferred LaTeX editor to generate a PDF report.

Create a LaTeX document with 3 clinical cases, where each page describes a clinical case with data from a pregnant woman and her newborn child. Include Apgar score data.

Certainly, here's an example LaTeX document with three clinical cases, each describing a pregnant woman and her newborn child, including Apgar score data. You can customize the content and formatting as needed:

latex Copy code

```latex
\documentclass{article}
\usepackage{geometry} % For adjusting page margins
\usepackage{lipsum}   % For generating placeholder text

% Adjust page margins if needed
\geometry{top=1in, bottom=1in, left=1in, right=1in}

\title{Clinical Cases Report}
\author{Your Name}
\date{\today}

\begin{document}

\maketitle

\section{Clinical Case 1}

\textbf{Patient Information:}

\begin{tabular}{ll}
\textbf{Name:} & Jane Doe \\
\textbf{Age:} & 28 years \\
\textbf{Gestational Age:} & 37 weeks \\
\end{tabular}

\textbf{Newborn Information:}

\begin{tabular}{ll}
\textbf{Name:} & Baby Doe \\
\textbf{Weight:} & 3.2 kg \\
\textbf{Apgar Scores:} & 1st Minute - 8, 5th Minute - 9 \\
\end{tabular}

\section{Clinical Case 2}

\textbf{Patient Information:}

\begin{tabular}{ll}
\textbf{Name:} & Sarah Smith \\
\textbf{Age:} & 32 years \\
\textbf{Gestational Age:} & 39 weeks \\
\end{tabular}

\textbf{Newborn Information:}

\begin{tabular}{ll}
\textbf{Name:} & Baby Smith \\
\textbf{Weight:} & 3.5 kg \\
\textbf{Apgar Scores:} & 1st Minute - 9, 5th Minute - 9 \\
\end{tabular}

\section{Clinical Case 3}

\textbf{Patient Information:}

\begin{tabular}{ll}
\textbf{Name:} & Emily Johnson \\
\textbf{Age:} & 25 years \\
\textbf{Gestational Age:} & 38 weeks \\
```

```
\end{tabular}

\textbf{Newborn Information:}

\begin{tabular}{ll}
\textbf{Name:} & Baby Johnson \\
\textbf{Weight:} & 2.8 kg \\
\textbf{Apgar Scores:} & 1st Minute - 7, 5th Minute - 8 \\
\end{tabular}

\end{document}
```

In this LaTeX document, we have three sections, each representing a clinical case. Each case includes patient information (name, age, and gestational age) and newborn information (name, weight, and Apgar scores at the 1st and 5th minutes). You can customize the patient and newborn information, add more details, or modify the formatting to suit your needs. Compile the document using your preferred LaTeX editor to generate a PDF report.

Taking into consideration the prepared materials (the dataset and clinical cases), propose a 2-hour lesson plan with the aim of a) presenting the importance of data quality in medicine, and b) promoting correct attitudes in this domain among future physicians. The target audience for the lesson is 4th-year medical students.

Certainly, here's a 2-hour lesson plan aimed at presenting the importance of data quality in medicine and promoting correct attitudes in this domain among fourth-year medical students. The plan is divided into several sections:

Lesson Plan: Understanding Data Quality in Medicine

Target Audience: Fourth-year medical students

Duration: 2 hours

Learning Objectives:

1. Understand the significance of data quality in medical practice.
2. Learn about the impact of data quality on patient care and research.
3. Promote proper data collection and recording practices among future physicians.

Introduction (15 minutes)

- Welcome students and provide an overview of the lesson.
- Explain the importance of data in medical practice.
- Highlight that medical decisions are increasingly data-driven.
- Introduce the connection between data quality and patient outcomes.

Discussion: The Role of Data in Medicine (20 minutes)

- Engage students in a discussion about the different types of data used in medicine (e.g., patient records, clinical trials, diagnostic tests).
- Discuss how data informs medical decisions, research, and policy.
- Mention the challenges associated with data management in healthcare.

Activity: Apgar Scores and Gestational Weeks (30 minutes)

- Present the synthetic dataset created earlier, emphasizing its importance in medical research.
- Divide students into small groups and provide each group with a subset of the dataset.
- Ask groups to analyze the dataset to find any patterns or correlations between Apgar scores and gestational weeks.
- Encourage discussions within groups.

Presentation: Importance of Data Quality (20 minutes)

- Discuss the critical role of data quality in medicine:
  - How data errors can lead to incorrect diagnoses and treatments.
  - The impact on research validity and reliability.
  - Legal and ethical implications.
- Provide real-world examples of data quality issues in healthcare.

Break (10 minutes)

- Allow students to take a short break.

Case Studies: Clinical Cases with Data (30 minutes)

- Present the clinical cases created earlier, emphasizing the importance of accurate data in patient care.

- Discuss the impact of incorrect or incomplete data on patient outcomes.
- Encourage students to analyze each case and identify potential data quality issues.

Group Discussion: Promoting Correct Attitudes (20 minutes)

- Lead a discussion on the importance of cultivating proper data collection and recording attitudes among future physicians.
- Discuss strategies to ensure data accuracy and completeness in medical practice.
- Encourage students to share their insights and experiences.

Activity: Data Quality Checklist (15 minutes)

- Provide students with a data quality checklist for medical records and research.
- Have students work individually or in pairs to review a sample medical record or research paper, applying the checklist to identify potential data quality issues.

Conclusion and Q&A (15 minutes)

- Summarize the key takeaways from the lesson.
- Open the floor for questions and engage in a Q&A session.
- Provide additional resources for students interested in learning more about data quality in medicine.

Assignment (Homework) (10 minutes)

- Assign a homework task that requires students to review a medical research paper and identify data quality issues.
- Set a deadline for submission.

Wrap-Up (5 minutes)

- Thank students for their participation and attendance.
- Remind them of the importance of data quality in their future medical careers.
- Provide contact information for further questions or concerns.

By the end of this lesson, fourth-year medical students should have a better understanding of the importance of data quality in medicine and be more conscious of the role they play in ensuring accurate and reliable data collection and recording practices.

Provide an example of rules that could be part of an obstetric clinical guideline based on gender, gestational week, Apgar scores at 1st and 5th minutes, and weight

Clinical guidelines in obstetrics are designed to provide evidence-based recommendations for healthcare providers to ensure the best care for pregnant individuals and their newborns. These guidelines typically take into account various factors, including gender, gestational week, Apgar scores at 1st and 5th minutes, and weight. Here's an example of rules that could be part of an obstetric clinical guideline:

Title: "Management of Neonates in the Delivery Room"

Rule 1: Apgar Score Assessment

- **Condition:** For all neonates delivered, assess the Apgar score at 1st and 5th minutes after birth.
- **Rationale:** Apgar scores provide a quick assessment of the neonate's overall condition and response to birth. This information helps determine the need for immediate medical intervention or further monitoring.

Rule 2: Apgar Score Interpretation

- **Condition:** Interpret Apgar scores as follows:
  - Apgar Score at 1st Minute:
    - 7-10: Considered normal.
    - < 7: May require immediate attention and possible resuscitation.
  - Apgar Score at 5th Minute:
    - 7-10: Indicates the neonate's continued adjustment to extrauterine life.
    - < 7: Suggests the need for further evaluation and intervention.
- **Rationale:** Interpreting Apgar scores at specific time points helps identify neonates at risk of neonatal distress or other medical issues, guiding clinical decisions.

Rule 3: Gestational Week Assessment

- **Condition:** Consider the gestational week when assessing neonatal health and potential complications.
- **Rationale:** Gestational age is a critical factor in determining neonatal maturity and potential risks associated with preterm or post-term births. It influences clinical management and interventions.

Rule 4: Gender-Specific Considerations

- **Condition:** Recognize that certain medical conditions or risks may be gender-specific.
- **Rationale:** Some medical conditions or risks, such as congenital anomalies, can be more prevalent in specific genders. Healthcare providers should consider gender-specific factors when evaluating and managing neonates.

Rule 5: Weight Assessment

- **Condition:** Measure and record the neonate's birth weight accurately.
- **Rationale:** Birth weight is an essential parameter for assessing neonatal health and potential complications. It helps identify low birth weight or macrosomic infants who may require special care.

Rule 6: Individualized Care Plans

- **Condition:** Develop individualized care plans for neonates based on their Apgar scores, gestational age, gender, and birth weight.
- **Rationale:** Every neonate is unique, and their care should be tailored to their specific needs and risk factors. Individualized care plans ensure the best possible outcomes.

These rules provide a foundation for managing neonatal care in the delivery room. Clinical guidelines in obstetrics should be evidence-based and regularly updated to reflect the latest research and best practices in the field. Additionally, they should consider ethical and cultural considerations to provide comprehensive care to pregnant individuals and their newborns.

Draw that tree using the DOT language.

Creating a tree diagram using the DOT language can be a bit complex in plain text, but I'll provide you with a simplified representation of the rules mentioned earlier. You can then use this as a starting point to create a more detailed diagram using a DOT language visualization tool.

Here's a simplified representation in DOT language:

dot Copy code

```dot
digraph ClinicalGuideline {
    // Rule 1
    "Assess Apgar Score at 1st and 5th Minutes" -> "Interpret Apgar Scores"

    // Rule 2
    "Interpret Apgar Scores" -> "Apgar Score at 1st Minute"
    "Interpret Apgar Scores" -> "Apgar Score at 5th Minute"

    // Rule 3
    "Consider Gestational Week" -> "Assessment Based on Gestational Age"

    // Rule 4
    "Recognize Gender-Specific Factors" -> "Consider Gender-Specific Conditi

    // Rule 5
    "Measure and Record Birth Weight" -> "Assessment Based on Birth Weight"

    // Rule 6
    "Develop Individualized Care Plans" -> "Consider Apgar Scores"
    "Develop Individualized Care Plans" -> "Consider Gestational Age"
    "Develop Individualized Care Plans" -> "Consider Gender"
    "Develop Individualized Care Plans" -> "Consider Birth Weight"
}
```

This DOT code represents a basic tree diagram with nodes and directional arrows representing the relationships between the rules in the clinical guideline. You can use a DOT language visualization tool like Graphviz to create a graphical representation of this tree diagram.
